# MicroED methodology and development

**DOI:** 10.1063/1.5128226

**Published:** 2020-02-13

**Authors:** Brent L. Nannenga

**Affiliations:** Chemical Engineering, School for Engineering of Matter, Transport, and Energy, Arizona State University, Tempe, Arizona 85287, USA and Center for Applied Structural Discovery, The Biodesign Institute, Arizona State University, Tempe, Arizona 85281, USA

## Abstract

Microcrystal electron diffraction, or MicroED, is a method that is capable of determining structure from very small and thin 3D crystals using a transmission electron microscope. MicroED has been successfully used on microcrystalline samples, including proteins, peptides, and small organic molecules, in many cases to very high resolutions. In this work, the MicroED workflow will be briefly described and areas of future method development will be highlighted. These areas include improvements in sample preparation, data collection, and structure determination.

## INTRODUCTION

In the last decade, crystallography has seen a dramatic reduction in the crystal size required for structure determination. Micro- and nanocrystals that were once considered too small to be of use are now delivering high-resolution data for a variety of samples, and this has all been due to the development and implementation of new crystallographic methods. Newer microfocused beamlines have allowed the collection of x-ray diffraction data from much smaller crystals than was possible from previous generation synchrotrons,^1^ and when coupled with serial crystallography, approaches are able to determine protein structures from crystals as small as 5 *μ*m.^2^ To access crystals of an even smaller size, serial femtosecond crystallography (SFX) with x-ray free-electron lasers (XFELs) can be used. This method uses extremely strong, yet very fast x-ray pulses, on the order of femtoseconds, to collect diffraction data before the crystals are destroyed by the high intensity x-ray pulse.^3^ In addition to these x-ray based microcrystallography methods, electron diffraction methods, including microcrystal electron diffraction, or MicroED, can be used for structure determination from extremely small crystals.^4^

While there are several benefits of using electron diffraction for microcrystal diffraction experiments, two key advantages of the technique relative to other x-ray based methods are the availability of instrumentation and the small number of crystals needed for structure determination. Electron diffraction experiments are conducted using cryo-electron transmission microscopes (cryo-TEMs) that are equipped with high-speed detectors. Because of cryo-electron microscopy's (cryo-EM) recent explosion in popularity for structural analysis of biological samples,^5,6^ cryo-TEM instrumentation capable of performing electron diffraction experiments is widespread and accessible. This is in direct contrast to the scarcity of XFEL facilities, which currently stands at only five facilities worldwide and makes obtaining beamtime for SFX microcrystal experiments difficult.^7^ Also, electron diffraction can make use of a very small number of crystals for structure determination, and in some cases, diffraction data from a single crystal is sufficient.^8–11^ This significantly reduces the amount of sample required to perform experiments, which is a great advantage when studying targets where sample quantity can be limiting, such as with difficult to express proteins or small molecules derived from natural sources. Primarily because of these factors, electron diffraction methods have grown in popularity for high-resolution structure determination from sub-micrometer thick crystals. Since the first protein structure of lysozyme was determined by MicroED in 2013,^12^ there have been 74 structures published in the Protein Data Bank (PDB) that have been determined from microcrystals using electron diffraction. Additional structures of small molecules have been deposited in other databases, such as the Cambridge Structural Database. Here, I will describe the procedure of MicroED data collection and data processing, and discuss recent advances and applications in electron diffraction for structure determination and opportunities for future developments.

### Electron crystallography

In the past, electron crystallography for biological samples was reserved for the analysis of 2D crystals, where each crystal was imaged or^13,14^ diffracted at single time.^15,16^ 2D electron crystallography provided some of the first structural data for membrane proteins,^17–22^ including the initial demonstration of the α-helices in bacteriorhodopsin span the lipid membrane.^23^ Thin 3D crystals of the biological material that consisted of more than a few layers were also analyzed and shown to produce diffraction data that could be assumed kinematic;^24^ however, structures were not able to be determined from these diffraction data. A complicating factor in the analysis of electron diffraction data from 3D microcrystals is that the crystal orientation is difficult to determine from a single diffraction pattern due to the flatness of the Ewald sphere.^25^ In order to collect multiple diffraction patterns from more beam sensitive materials (e.g., biomolecular crystals) and facilitate structure determination from these samples in a similar manner as above, the MicroED method uses cryogenic temperatures in a cryo-TEM and collects electron diffraction data as the crystals are continuously rotated in the beam under very low doses.^10^ The continuous rotation of the crystal in the beam, which was inspired by the way data are collected in X-ray crystallography, was a unique advance with the introduction of MicroED. The combined effects of low-doses, cryogenic temperatures to reduce radiation damage and high-speed and sensitive detectors facilitate the structure determination of samples such as proteins, peptides, and radiation sensitive organic molecules. This limitation of microcrystal data collection was also overcome for less beam sensitive samples by taking a series of diffraction patterns as the stage is rotated in discrete steps, which can also be coupled with beam tilting or beam precession.^26–31^ Because the tilt angles between diffraction patterns are known, the relationship between each diffraction pattern can be established and the unit cell parameters and orientation of the crystal can be determined. These methods, known as automated diffraction tomography (ADT),^26^ rotation electron diffraction (RED),^31^ or precession-assisted diffraction tomography (PEDT),^30^ have been used to determine the structures from many samples that resisted structure determination by other methods. These micro- and nanocrystalline samples include, but are not limited to, minerals,^32–34^ advanced materials,^35,36^ zeolites,^37–39^ and metal–organic frameworks.^40,41^ For these structure analyses, diffraction data are either collected at ambient or cryo-temperature. If necessary, vitrified samples are used.

## MICROED DATA COLLECTION AND PROCESSING

Detailed protocols on MicroED sample preparation and data collection have been previously described, and readers are encouraged to refer to these publications for further guidance.^12,42–44^ Samples for MicroED analysis are prepared on EM grids similar to standard cryo-EM sample preparation procedures used for single particle cryo-EM. Once loaded into the cryo-TEM, the first task is to screen the grid at low magnification to identify promising areas of the grid. The beam will not penetrate the sample in regions where the vitrified buffer is too thick, and these thick areas are to be avoided on the grid. Other areas of the grid that should be ruled out during this low magnification screening stage are areas where the carbon film has been significantly broken, or where very large crystals are covering the majority of the visible area in a grid square. Once suitable areas have been identified at low magnification, the search magnification is increased to a level where the field of view covers approximately one grid square of the EM grid. It is imperative that this searching be done at extremely low doses (e.g., using over-focused diffraction mode or low magnification imaging modes) so that the crystals do not experience significant radiation damage during screening. During the medium magnification searching, promising crystals are identified based on the size and shape. Initial diffraction patterns are collected by first centering the crystal of interest in the beam in the search mode. The crystal is then exposed to the beam by switching the mode of the cryo-TEM to diffraction, and the quality of this first diffraction pattern is judged based on the diffraction resolution and quality of the reflections (sharp and well-separated single spots on the detector). When a quality crystal is identified, rotation diffraction datasets are collected. Parameters that need to be optimized for each sample are rotation speed of the stage and integration time on the detector. These two parameters will combine to set the angular wedge of data that will be collected per frame, and it is important that spots do not begin to overlap as the rotation angle per frame is increased. At the general starting point, we collect 0.5–1.0 degree per frame rotations for proteins and 1.0–2.0 degree per frame rotations for peptides and small molecules. These parameters are typically optimized for each individual sample. This is accomplished by first tilting the cryo-TEM stage to a high angle. If the eucentric height of the stage is set correctly, the crystal should not move during the rotation, and this should be confirmed by watching the crystal rotation in the search mode prior to data collection. If the stage of the TEM does not allow the precise centering of the crystal during the entire tilt range, automated crystal tracking procedures can also be employed to track the crystal during data collection and recenter it in the beam;^26,45^ however, this required additional microscope control software than what is discussed here. For both screening and data collection, the diffraction area is controlled either through the use of the selected area aperture or by limiting the size of the beam. This helps us to reduce the background noise by ensuring that data are only collected from the crystal and not from the surrounding area.

Because the rotation of the crystals in the beam is continuous, the diffraction data are collected using a fast detector to avoid significant gaps in the data while the detector is being readout. There are many cameras that have been used to collect electron diffraction data and are either high-speed complementary metal-oxide semiconductor (CMOS) detectors,^10,12,46,47^ direct electron detectors,^48,49^ or hybrid pixel detectors.^50–52^ Each of these different detector types have their strength and weaknesses (e.g., large number of pixels and ability to also image for CMOS detectors and zero readout noise for hybrid pixel detectors), and it will be important for the diffraction community and detector manufacturers to identify which cameras have the best performance and to continue to improve upon each design.

## DATA PROCESSING AND STRUCTURE REFINEMENT

Because MicroED data are collected as the crystal is continuously rotated in the electron beam, the final datasets resemble those collected by conventional x-ray diffraction experiments. Therefore, crystallographic data processing programs that have been developed previously for x-ray crystallography can be easily used to process electron diffraction data. The first stage of MicroED data processing is that the data are converted to file formats which can be read by the data processing programs.^47^ It is important to ensure that the unique aspects of electron diffraction geometry are described, such as the wavelength (much smaller than x-ray experiments) and the camera length (much longer than x-ray experiments). The wavelength of the experiment can be derived from the accelerating voltage of the electron microscope. Because the camera length in an electron diffraction experiment is not a physical detector distance, and because the camera length displayed on the microscope is not the true distance, it is critically important that the camera lengths be calibrated prior to MicroED data collection. This can be accomplished by using calibration grids containing polycrystalline standard samples (e.g., gold or evaporated aluminum).

Once the converted diffraction data are loaded into the data processing programs of the user's choice, data indexing and integration are followed using relatively routine procedures for those programs. Several data processing programs have been successfully employed, including XDS,^53^ MOSFLM,^54,55^ and DIALS.^56,57^ When the PDB is searched for structures that have been deposited into the data bank that have used for electron crystallography on 3D crystals, the most popular data processing programs by far have been XDS and MOSFLM. MOSFLM, which has been used on approximately 20% of all PDB depositions, was the first data processing program used on continuous rotation data. The first use of MOSFLM and continuous rotation ultimately yielded a 2.5 Å structure of lysozyme,^10^ and since that time MOSFLM has mainly been used on the processing of MicroED data from protein crystals. It is important to note that the diffraction datasets for this 2.5 Å lysozyme structure extended to higher resolution, and the data processing set the ultimate resolution of this structure. Subsequent improvements to data processing have allowed similar crystals of lysozyme to yield structures at higher resolution.^58^ Currently, XDS is the most popular data processing program for MicroED data with over 65% of all electron diffraction structures in the PDB having used XDS. One of the benefits of XDS is that it works well with protein samples as well as peptides and small molecules. If small molecule structures are included with those deposited in the PDB, the relative usage and popularity of XDS are increased even more.

**FIG. 1.**
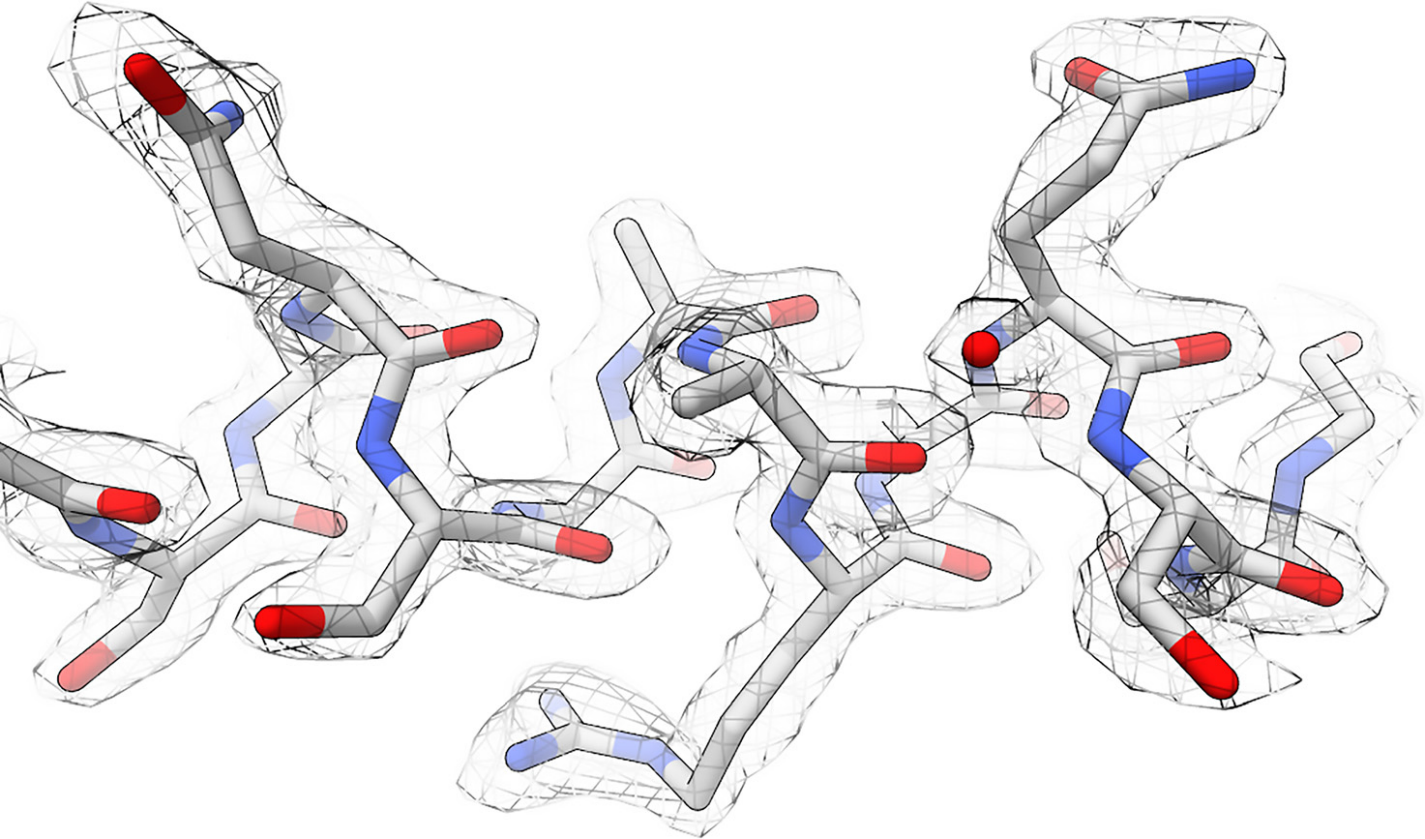
Representative density map surrounding a segment of Proteinase K. The structure was determined to 2.0 Å resolution, and the density map in the image is contoured at 1.5σ.^112^

MicroED data are phased either by molecular replacement (using programs such as Phaser^59^ or MOLREP^60^) or by direct methods (e.g., SHELXT,^61^ SIR^62^) or by hybrid iterative algorithms (e.g., SUPERFLIP^63^) if the resolution of the data is sufficient as is often the case with peptides or small molecules.^64^ Following phasing, protein structures are refined using commonly used refinement x-ray crystallographic programs such as phenix.refine^65^ and REFMAC.^66^ The use of electron scattering factors is available in both of these programs, as should be used when refining models against MicroED data as it improves the quality of the resulting density maps as well as the R-factors. The final density maps obtained from MicroED, which are the maps of the coulombic potential rather than the electron density, are very high-quality and similar to what would be seen with data from x-ray crystallography^67^ (Fig. 1). For small molecules and samples from materials science, other programs can be used for structure refinement such as SHELXL^68^ and JANA2006.^69^

When electron diffraction data are collected by other methods that produce data which are not analogous to MicroED and X-ray diffraction data, other programs specifically developed for these methods can be used for electron diffraction data processing and refinement. These programs include, but are not limited to, ADT3D,^70^ RED data processing,^31,71^ and PETS.^72^

## APPLICATIONS OF MicroED

The initial work on structure determination by MicroED was focused on the analysis of protein microcrystals, and the first paper in 2013 represented the determination of structure by electron diffraction data collected from 3D microcrystals for the first time.^12^ Since this time many protein structures have been determined,^4,11,73^ including a recent protein structure that could not be determined by other methods.^74^ In addition to the study of protein structure, the application of MicroED to the study of peptides, specifically peptides involved in neurodegenerative diseases, has been incredibly successful.^64,75–82^ The first application of MicroED to the study of peptides was published in 2015 and represented the determination of a novel structure from a biomolecule for the first time.^81^ This study determined the structure of an 11-residue segment of α-synuclein, the protein involved in Parkinson's disease, that is responsible toxicity associated with aggregation. These extremely small crystals had resisted many other structure determination methods, including the use of XFELs, however, the use of MicroED facilitated rapid structure determination of these important peptides. Since this initial study, many other peptides have been analyzed by MicroED leading to improved understanding of protein aggregation, peptide crystal structure, peptide biosynthesis, and structure-based therapeutics.^48,75,78,82,83^

**FIG. 2.**
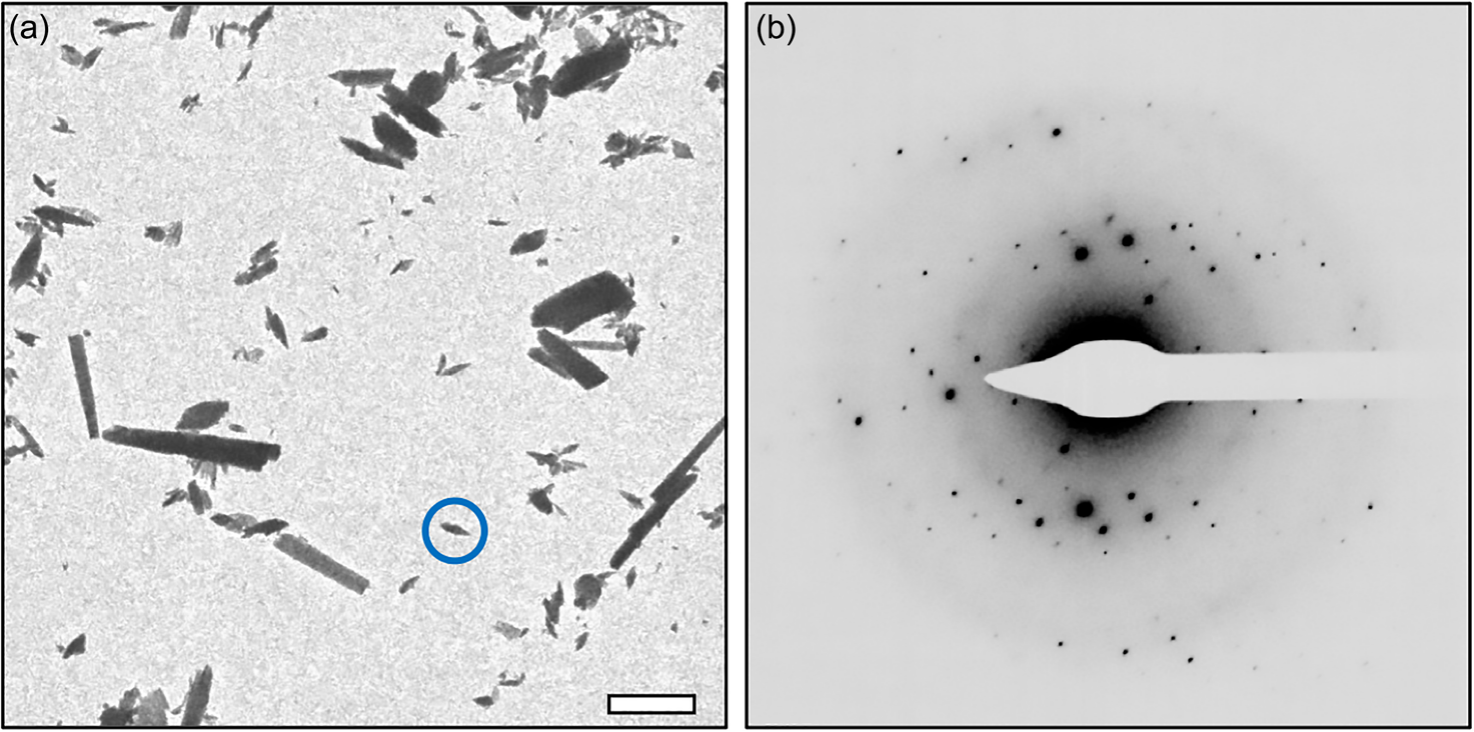
MicroED analysis of small organic molecules. (a) Small molecule crystals are deposited directly on the carbon coated EM grid, and diffraction data can be collected from the crystals. (b) Diffraction pattern collected from the small crystal circled in panel (a) shows that high-quality data can be collected even from extremely small crystals. The scale bar in panel (a) represents 2 *μ*m, and the edge of the detector in panel (b) is approximately 0.65 Å. Diffraction data were collected on a Titan Krios cryo-TEM equipped with a CETA D camera for diffraction data collection.

An application of electron diffraction which has been generating a great deal of interest recently is for small molecule structure determination.^50,52,84^ MicroED for small molecules adds a powerful method to the study and synthesis of organic compounds. In many cases, synthesized materials can be directly applied to the EM grid, and this powder material will actually contain many small micro- and nanocrystals [see, for example, Fig. 2(a)]. The amount of material needed to prepare the grids is extremely small (much less than a milligram) so that MicroED can be used on samples where the quantity available would generally preclude structural studies. This approach uses one of MicroED's greatest advantages, the fact that the user is able to directly visualize the nano-/microcrystals on the grid and choose the best crystals to collect data from. Even very small nanocrystals are capable of producing very high-quality and high-resolution diffraction data [Fig. 2(b)], which can be used for structure determination. In one study, ten small molecule structures were determined very quickly (approximately 3 min for data collection and just a few minutes for initial structure determination) directly from powdered samples.^84^ The study also demonstrated that a heterogeneous sample containing four different compounds could be used for MicroED analysis. All four compounds in the mixture could be easily identified by unit cell, and data could be collected from each of the crystals for structure determination. In another study, rapid data collection and structure determination were used to determine the structure of a new small molecule derivative as well as the structure of a pharmaceutical directly from a pill obtained from a pharmacy.^52^ In all these cases, the crystals used for structure determination were several orders of magnitude smaller than what would be required for single crystal x-ray diffraction experiments. This demonstrates the outstanding potential for MicroED in the area of small molecule, pharmaceutical, and natural products research.

## ADVANCES IN METHODOLOGY AND FUTURE PERSPECTIVES

When preparing microcrystalline protein samples for MicroED, one of the more time-consuming steps is the preparation of quality samples for diffraction data collection. The sample handling procedures described above work well for many samples; however, for samples, where the mother liquor of the crystals is difficult to blot away, or where the crystals are too thick for the collection of diffraction data, new methods of sample preparation are needed. The fragmentation of larger crystals into smaller microcrystals has been shown to be a successful method for preparing MicroED samples.^85^ In this work, several methods for fragmentation were reported, including gentle sonication or vortexing, and the crystals processed by these methods could be used to determine high-resolution structures by MicroED, whereas the initial larger crystals did not yield x-ray data that could be used for structure determination. Also, crystal growth could potentially be stopped before the crystals have a chance to grow beyond what is useful for electron diffraction. An alternative method for processing crystals is the use of cryo-focused ion beam (cryo-FIB) milling, which uses a focused beam of ions to progressively machine thicker crystals into very thin lamella of only a few hundred nanometers. The grids containing the milled crystals can be transferred from the cryo-FIB into a cryo-TEM for MicroED data collection and structure determination. Cryo-FIB milling of crystals promises to greatly improve sample preparation by MicroED by facilitating the use of thicker crystals and samples within viscous media that is difficult to blot thin enough. Thus far, cryo-FIB milling has been reported on lysozyme^86,87^ and proteinase K,^88^ and it will be important that the procedures be validated on other more sensitive samples. Additionally, there are a limited number of cryo-FIB instruments available, and the time it takes to process samples reduces the otherwise high-throughput nature of MicroED. Increasing instrument availability and optimizing the processing procedures will be important in the future to allow the widespread use of cryo-FIB sample processing for MicroED.

Another issue that commonly arises while collecting data is the problem of preferred orientation of the crystals on the grid. When crystals have preferred orientation on the grid, the regions of reciprocal space that can be sampled is limited by how much the microscope stage can tilt (approximately ±70°). This is not a new problem to the field of electron crystallography as datasets collected from 2D crystals have always suffered from a missing wedge of data.^89^ The problem of preferred orientation is not as severe in MicroED as it is in 2D crystallography as the 3D shape of the crystals in some cases helps facilitate multiple orientations. Nevertheless, for some samples (e.g., plate-like crystals^8^), 3D microcrystals still have preferred orientation on the grid which leads to low completeness of the final dataset. This does not preclude structure determination but does reduce the final quality of the maps and model. Recently, EM girds with structure supports have been used to help alleviate the problem with preferred orientation,^90^ and the use of these grids were able to greatly increase the completeness of the data collected from a model zeolite sample.

High-throughput approaches to collecting MicroED data promises to increase the amount of data that can be collected in a single data collection session. Recently, SerialEM, a data collection program developed for the collection of cryo-EM images,^91^ was employed for the automated collection of MicroED data.^92^ Following the initial setup of the software and location of crystals on the grid, this approach allowed over 300 continuous rotation MicroED datasets to be collected overnight. The use of SerialEM for MicroED is attractive because many cryo-EM users are familiar with SerialEM, and the microscope setup is the same with SerialEM as it is used for standard MicroED data collection. In the future, this data collection strategy should be coupled with newly automated MicroED data processing methods in order to increase the throughput of the data processing, thereby enhancing the speed of the entire MicroED workflow. In another report, new software was developed to control the electron microscope and collect high-throughput continuous rotation data, and this software also included procedures for data analysis as the diffraction data were collected.^93^ In other work, a new integrated system data collection system with a graphical user interface was reported that allows high-quality data collection.^94^ Recently, serial crystallography approaches have been used to collect thousands of still diffraction patterns from nanocrystals on an EM grid.^95^ Following indexing and merging using programs developed for serial x-ray crystallography, high resolution structures were able to be determined with statistics similar to rotation datasets.

A key area of future research for MicroED is in employing and developing phasing methods. Currently, the only options for phasing MicroED data are using direct methods or iterative algorithms for high-resolution data or molecular replacement. Molecular replacement is an extremely popular phasing method and is used in many crystallographic experiments.^96^ However, in the cases where molecular replacement is not possible and the resolution is not sufficient for direct methods, new methods of phasing are needed for MicroED. If resolution is limited for small molecules, it has been shown that simulated annealing methods can be used for phasing and eventual structure solution.^97^ With electrons, there is no anomalous signal as there is when collecting x-ray diffraction data; therefore, phasing methods that rely on anomalous differences are not applicable to electron diffraction data. Isomorphous replacement methods should be applicable to MicroED data; however, to date, these methods have yet to be used for phasing. Imaging is an attractive alternative to solving the phase problem, and it has been successfully used for 2D crystallography,^15,16^ but as with isomorphous replacement imaging has yet to be used for MicroED data.

There are two issues that impact structure refinement in MicroED and improving the refinement of the models against the electron diffraction data is a critical area of development in MicroED. The first is the issue of dynamic scattering seen in electron diffraction, which becomes more problematic as the thickness of the crystal increases.^98^ The effects of dynamic scattering can be reduced by continuous rotation of the stage during data collection^10^ or by beam precession,^99^ and these are the approaches commonly used for diffraction data collection. While, in many cases, dynamic scattering is not significant enough to preclude structure determination, it still represents a source of error. Properly treating and refining dynamic scattering effects can improve the final results from electron diffraction data, revealing subtle structural differences.^100^ Significant work has been performed on handling multiple scattering for small organic molecules and inorganic materials. For example, when dynamical refinement was applied to microcrystals of paracetamol and a cobalt aluminophosphate framework, it led to the clear visualization of the hydrogen atoms in the molecules.^101^ By incorporating dynamic scattering refinement, it was also shown that electron diffraction can be used to determine the absolute structure of a pharmaceutical compound, highlighting the power of modeling dynamical effects.^102^ Recently, a method for correcting the dynamic scattering has been proposed,^103^ and when applied to lysozyme diffraction data, this approach was able to reduce the crystallographic R factors by a few percent. A second issue with refining models from MicroED data could potentially arise from the scattering factors used in refinement. In contrast to x-ray diffraction, electron diffraction is highly sensitive to chemical bonding and charge effects within the crystal, especially at lower resolutions, because of the very large changes in electron scattering factors at low angles due to bonding effects, compared to very small effects in x-ray diffraction.^104–108^ Because the scattering factors used for refinement of MicroED data retain some of the assumptions used for x-ray scattering (e.g., spherical shape), there are inaccuracies between the calculated and observed intensities. Attempts have been made in biological electron crystallography to model charge or bonding and thereby improve the final maps and models of biological molecules.^104,105,109–111^ Generally, these approaches have had only modest effects because they have focused on a few types of atoms or have been limited to small fragments. However, the modest effects seen could also be a result of overestimating the effects of bonding and charge; therefore, more investigation into these effects and assumptions is required. New approaches that would allow the efficient and accurate modeling electron scattering factors could help answer these questions. If these improved scattering factors could eventually be integrated into standard crystallographic refinement pipelines, they would be extremely valuable for improving final structures obtained by electron diffraction.

Electron crystallography of 3D micro- and nanocrystals has proven itself to be a valuable tool for high-resolution structure determination, and further development of the methodology promises to continue to drive the method forward. There are many groups around the world working on these methods and driving the field forward, with new advances coming at a very rapid pace. As described above, improved sample handling strategies, data collection methods, and advanced refinement procedures will continue to spread the use of MicroED and allow the structural analysis of new targets. It will be exciting to see how, in the future, these methods are able to employ both alone and in complement with other methods, such as serial femtosecond crystallography,^7^ to yield exciting new structural insights.
